# Assessment of the optical and electrical properties of light-emitting diodes containing carbon-based nanostructures and plasmonic nanoparticles: a review

**DOI:** 10.3762/bjnano.12.80

**Published:** 2021-09-24

**Authors:** Keshav Nagpal, Erwan Rauwel, Frédérique Ducroquet, Protima Rauwel

**Affiliations:** 1 Institute of Technology, Estonian University of Life Sciences, Kreutzwaldi 56/1, 51014 Tartu, Estonia; 2 Université Grenoble Alpes, IMEP-LaHC, 38016 Grenoble, France

**Keywords:** carbon nanotubes (CNT), graphene, light-emitting diodes (LED), plasmonic nanoparticles, quantum dots

## Abstract

Light-emitting diodes (LED) are widely employed in display applications and lighting systems. Further research on LED that incorporates carbon nanostructures and metal nanoparticles exhibiting surface plasmon resonance has demonstrated a significant improvement in device performance. These devices offer lower turn-on voltages, higher external quantum efficiencies, and luminance. De facto, plasmonic nanoparticles, such as Au and Ag have boosted the luminance of red, green, and blue emissions. When combined with carbon nanostructures they additionally offer new possibilities towards lightweight and flexible devices with better thermal management. This review surveys the diverse possibilities to combine various inorganic, organic, and carbon nanostructures along with plasmonic nanoparticles. Such combinations would allow an enhancement in the overall properties of LED.

## Review

### Introduction

Nanomaterials have engendered the miniaturization of devices, bringing about advances in a variety of fields, such as biomedicine, environmental technologies, optoelectronics, and photocatalysis [1–2]. In particular, light-emitting diodes (LED) have shown the most significant technological progress in display applications. They are incorporated into smart phones, TVs, traffic signals, and medical devices [3]. Similarly, lighting systems employing LED have longer life times (>50000 h), much lower heating effects, ultrafast response times, and a wider choice of emission wavelengths compared to conventional lighting systems.

Inorganic LED consist of inorganic semiconductor materials in the active region, for example thin films of GaAs that emit in the red to near-infrared (>700 nm) region [4]. Ga-based LED belong to the III–V group of semiconductors and emit from the UV to the red region of the visible spectrum via bandgap tuning (i.e., on alloying with In and Al [5–7]). Similarly, other active materials for quantum dot light-emitting diodes (QLED), such as the II–VI semiconductor family include ZnO, CdSe, CdS, CdTe, ZnSe, ZnS, ZnTe, and their core–shell structures with Zn-based compounds possessing higher bandgaps than Cd-based compounds [8–12]. The wide bandgap of Zn-based compounds has provided an opportunity to produce blue-emitting ‘all ZnO’-based LED, following the successful fabrication of p-type ZnO [13].

Organic light-emitting diodes (OLED) possess several interesting properties and are therefore gaining popularity [14]. Their low-cost and facile fabrication routes, wider viewer angle, higher resolution, lower-power consumption, lightweight, higher contrast, and faster switching characteristics give them leverage over inorganic LED in display applications. Organic light-emitting diodes (OLED) consist of photoactive polymers, such as PPV and MEH-PPV that can be deposited as highly ordered crystalline thin films [15–16]. Despite several advantages, OLED have certain drawbacks, such as lower lifetime, high cost, early degradation, and a low overall performance including poor external quantum efficiencies (EQE) as compared to inorganic LED. A combination of LED and OLED or hybrid LED (HyLED) overcame some of these drawbacks. Nevertheless, HyLED presently may not be the most popular LED for the display market as they suffer from energy losses due to total internal reflection at the emitter/air interface.

Several methods have been proposed to tune the properties of LED. To that end, multiwall carbon nanotubes (MWNT) and single-wall carbon nanotubes (SWNT) have been applied to various layers in LED, such as the emissive layer (EML), the hole transport layers (HTL), the electron transport layers (ETL), the cathode, and the anode [17–21]. Enhancement in LED properties via surface plasmon resonance (SPR) of metal nanoparticles (MNP) such as Au and Ag have also been reported [22–23]. This manifests as an increment in the photoluminescence (PL), conductivity, and electroluminescence (EL) of the LED [24–25]. Other MNP with SPR properties include Al, Pt, Pd, and Cu [26–29]. In general, SPR not only increases the radiative recombination lifetime values, but also the quantum yield whereupon the luminous and internal quantum efficiencies of the device increase. However, SPR is very sensitive to the shape and size of MNP, which in turn directly influence the overall properties of LED.

This manuscript reviews the effect of carbon nanostructures, such as carbon nanodots, carbon nanotubes (CNT), and graphene (GR) towards producing cost-effective and efficient LED. A second strategy consisting of enhancing the optical and electrical properties of LED via SPR of MNP is also surveyed. The LED covered in this review include inorganic LED, OLED, inorganic/organic LED, and HyLED. The feasibility of incorporating GR and CNT in large-scale devices is also discussed. Even though plasmonic nanoparticles (NP) are a developing field, their applications are nonetheless promising. The effect of these nanostructures on the performance of LED when included in individual layers (i.e., anode, HTL, EML, ETL, and cathode) is analyzed. Subsequently, various characteristics of LED containing carbon nanostructures and plasmonic NP are discussed in terms of EQE, internal quantum efficiency, luminance, EL, and current–voltage (*I*–*V*) characteristics. A list of various abbreviations employed in this review is available in Table 1.

**Table 1:** List of abbreviations mentioned in this review.

Abbreviation	Full name

CNT	carbon nanotubes
CQD	carbon quantum dots
EBL	electron blocking layer(s)
EIL	electron injection layer(s)
EML	emissive layer(s)
EL	electroluminescence
ETL	electron transport layer(s)
EQE	external quantum efficiency
FTO	fluorine-doped tin oxide(s)
HBL	hole blocking layer(s)
HIL	hole injection layer(s)
HOMO	highest occupied molecular level
HTL	hole transport layer(s)
HyLED	hybrid light-emitting diode(s)
IFOLED	inverted fluorescent OLED
IPOLED	inverted phosphorescent OLED
ITO	indium tin oxide(s)
LED	light-emitting diode(s)
LUMO	lowest unoccupied molecular level
MNP	metal nanoparticles
MWNT	multiwall carbon nanotubes
NP	nanoparticles
OLED	organic light-emitting diode(s)
PL	photoluminescence
QD	quantum dots
QLED	quantum dot light-emitting diode(s)
SACNT	super-aligned carbon nanotubes
SWNT	single-wall carbon nanotubes
TCO	transparent conducting oxide(s)

### Enhancing the anode characteristics

For LED, the general strategy is to use a current-spreading layer (anode) with a high electrical conductivity and a high transparency ranging from the UV to the red region. Additionally, it should also be cost-effective and producible on a large scale. For this purpose, transparent conducting oxides (TCO), such as thin films of In_2_O_3_, SnO_2_, ZnO, and their mixtures have been extensively studied [30–34]. The most widely studied TCO is indium tin oxide (ITO), which possesses good physical properties, such as high optical transmittance (>80% in the visible region) and low sheet resistance (≈20 Ω/sq). Typically, ITO consists of 90% In_2_O_3_ and 10% SnO_2_ by weight. Kim et al. have grown ITO on glass substrates with varying Sn concentrations [35]. Although ITO is a conventional favorite current-spreading layer, it nevertheless suffers from certain limitations, including high processing temperatures, cracking upon bending, and poor transparency in the blue and UV regions. In addition, ITO is expensive owing to the fact that it is deposited by high-vacuum thin-film deposition methods and the price of indium has escalated by almost 900% since the last decade. These disadvantages can be surmounted by the addition of a current-spreading layer composed of carbon-based nanomaterials, such as GR and CNT [36].

**Figure 1:**
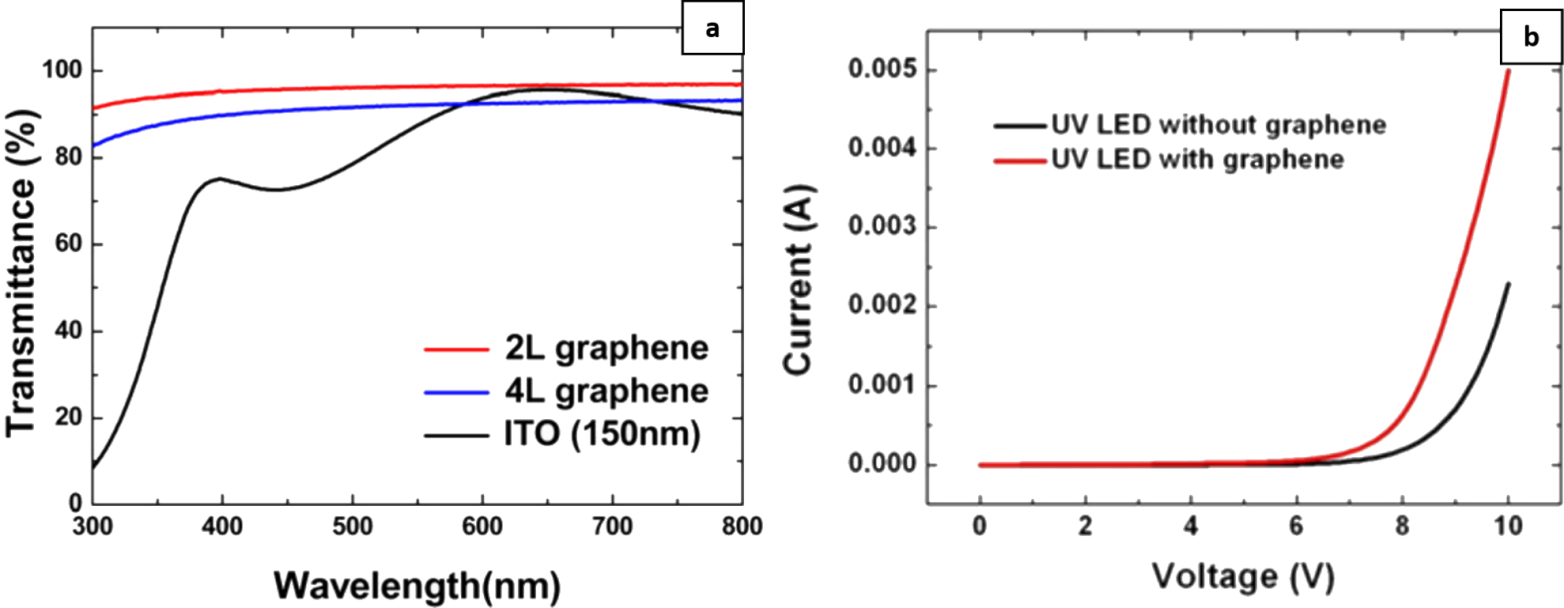
(a) Transmittance from 300–800 nm of ITO (150 nm), two-layer (2L) and four-layer (4L) graphene and (b) the *I*–*V* characteristics of UV LED with and without few-layer graphene-based conductive electrodes. Figure 1a and Figure 1b were adapted from [39], with the permission of AIP Publishing. This content is not subject to CC BY 4.0.

Carbon-based nanostructures play a dual role at the anode. Light-emitting diodes are self-heating, current-sensitive, and luminously intensive light sources. They are also highly dependent on ambient temperatures. The lifetime of LED, in particular OLED, is on an average reduced by 30–50% for each 10 °C rise in temperature. Further, self-heating in LED causes degradation of the active region, which further affects the efficiency and the operational lifetime of LED. Therefore, in order to obtain adequate performances from LED, proper thermal management of the device is required. In this regard, graphene at the anode tends to alleviate self-heating issues as it disperses the heat away from the active layer. This in turn also reduces the thermal resistance between the device layers [37–38]. Furthermore, the transmittance of large-area few-layer graphene used as a current-spreading layer in InGaN-based UV LED is similar to the transmittance of ITO in the blue region (Figure 1a) [39]. Furthermore, the *I*–*V* results in Figure 1b show an increase of the maximum current value from 2.3 to 5 mA at 10 V, when graphene is present.

**Figure 2:**
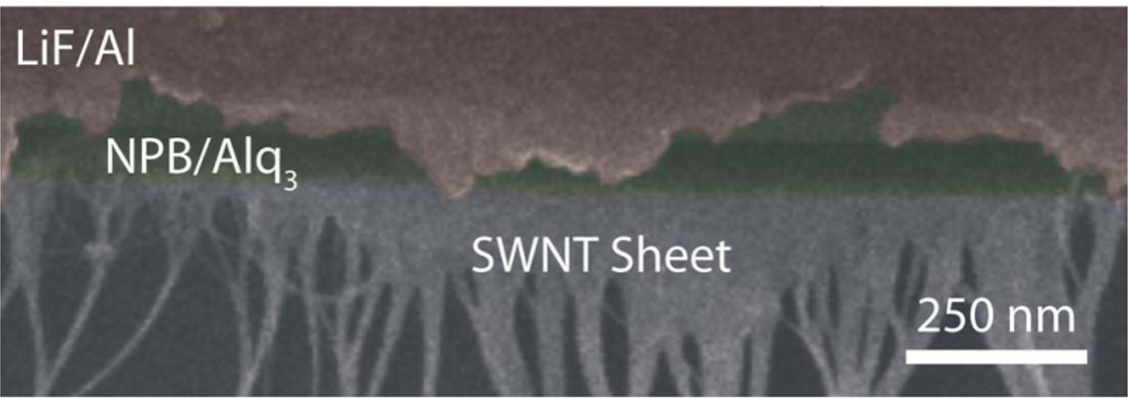
Cross-sectional scanning electron microscopy (SEM) image of the layers of the OLED. The various layers in the device are clearly visible including the vertical SWNT sheet. Figure 2 was adapted from [21], with the permission of AIP Publishing. This content is not subject to CC BY 4.0.

Guo et al. also reported an increase of about 40% in the EL intensity under a 5 mA current injection at room temperature in AlGaInP LED, after the deposition of graphene onto the anode or the GaP surface [40]. Roll-to-roll techniques and chemical vapor deposition, both industrially viable techniques, are capable of producing 30 inch wafers of graphene, thereby demonstrating the viable upscaling of its production [41]. Other carbon-based nanomaterials such as SWNT have also been employed as current-spreading layers. The optoelectronic properties of SWNT thin films make them ideal for transparent conducting flexible electrodes in LED. In Figure 2, Aguirre et al. pre-fabricated vertical sheets of SWNT and then transferred them onto a glass substrate [21]. The maximum brightness and efficiency achieved in these SWNT-based devices are 2800 cd/m^2^ and 1.4 cd/A, respectively, at a turn-on voltage of 6.6 V. These values are however not as satisfactory as the ITO-based device: 6000 cd/m^2^ and 1.9 cd/A at a turn-on voltage of 6.2 V. One reason for lower SWNT device performance is associated with its lower transmittance of 44% compared to 90% of ITO. A higher transmittance is nonetheless possible by optimizing the interface between HTL and SWNT via a parylene layer, which tends to increase the transparency of SWNT. Table 2 lists the polymers and their acronyms mentioned in this study.

**Table 2:** List of polymers and their acronyms mentioned in this review.

Acronyms	Full name

Alq_3_	tris(8-hydroxyquinoline)aluminum
BCHA-PPV	poly[2,5-bis(cholestanoxy) phenylene vinylene]
CuPc	copper(II) phthalocyanine
DCM	4-(dicyanomethylene)-2-*tert*-butyl-6-[2-(1,1,7,7-tetramethyljulolidin-4-yl)vinyl]-4*H*-pyran
F8BT	poly(9,9-dioctylfluorene-*alt*-benzothiadiazole)
HAT-CN	1,4,5,8,9,11-hexaazatriphenylenehexacarbonitrile
MEH:PPV	poly[2-methoxy-5-(2'-ethylhexyloxy)-1,4-phenylene vinylene]
M3EH-PPV	poly[2,5-dimethoxy-1,4-phenylene-1,2-ethenylene-2-methoxy-5-(2-ethylhexyloxy)−(1,4-phenylene-1,2-ethenylene)]
MDMO-PPV	poly(3',7'-dimethyloctyloxy phenylene vinylene)
NPB	*N*,*N*'-di(1-naphthyl)-*N*,*N*'-diphenyl-(1,1'-biphenyl)-4,4'-diamine
NSD	*N*-(3-aminopropyl)propane-1,3-diamine
PBD	2-(4-biphenyl)-5-(4-*tert*-butylphenyl)-1,3,4-oxadiazole
PBD-PMMA	2‐(4‐biphenyl)‐5‐(4‐*tert*‐butylphenyl)‐1,3,4‐oxadiazole in poly(methyl methacrylate)
PEDOT:PSS	poly(3,4-ethylenedioxythiophene) polystyrene sulfonate
PET	polyethylene terephthalate
PFO	polydioctylfluorene
PmPV	poly[(*m*-phenylene vinylene)-*co*-(2,5-dioctoxy-*p*-phenylene vinylene)]
PMMA	poly(methyl methacrylate)
PPV	poly(*p*-phenylene vinylene)
PPE-PPV	poly(2,5-dialkoxy-1,4-phenylene ethynylene)
PVK	poly(9-vinylcarbazole)
spiro-OMeTAD	2,2',7,7'-tetrakis[*N*,*N*-di(4-methoxyphenyl)amino]-9,9'-spirobifluorene
TAD	1,2,4-triazoline-3,5-dione
TCTA	4,4',4-tris(carbazol-9-yl)triphenylamine
TmPyPB	1,3,5-tri(*m*-pyridin-3-ylphenyl)benzene
TPD	poly(*N*,*N*'-bis-4-butylphenyl-*N*,*N*'-bisphenyl)benzidine

**Figure 3:**
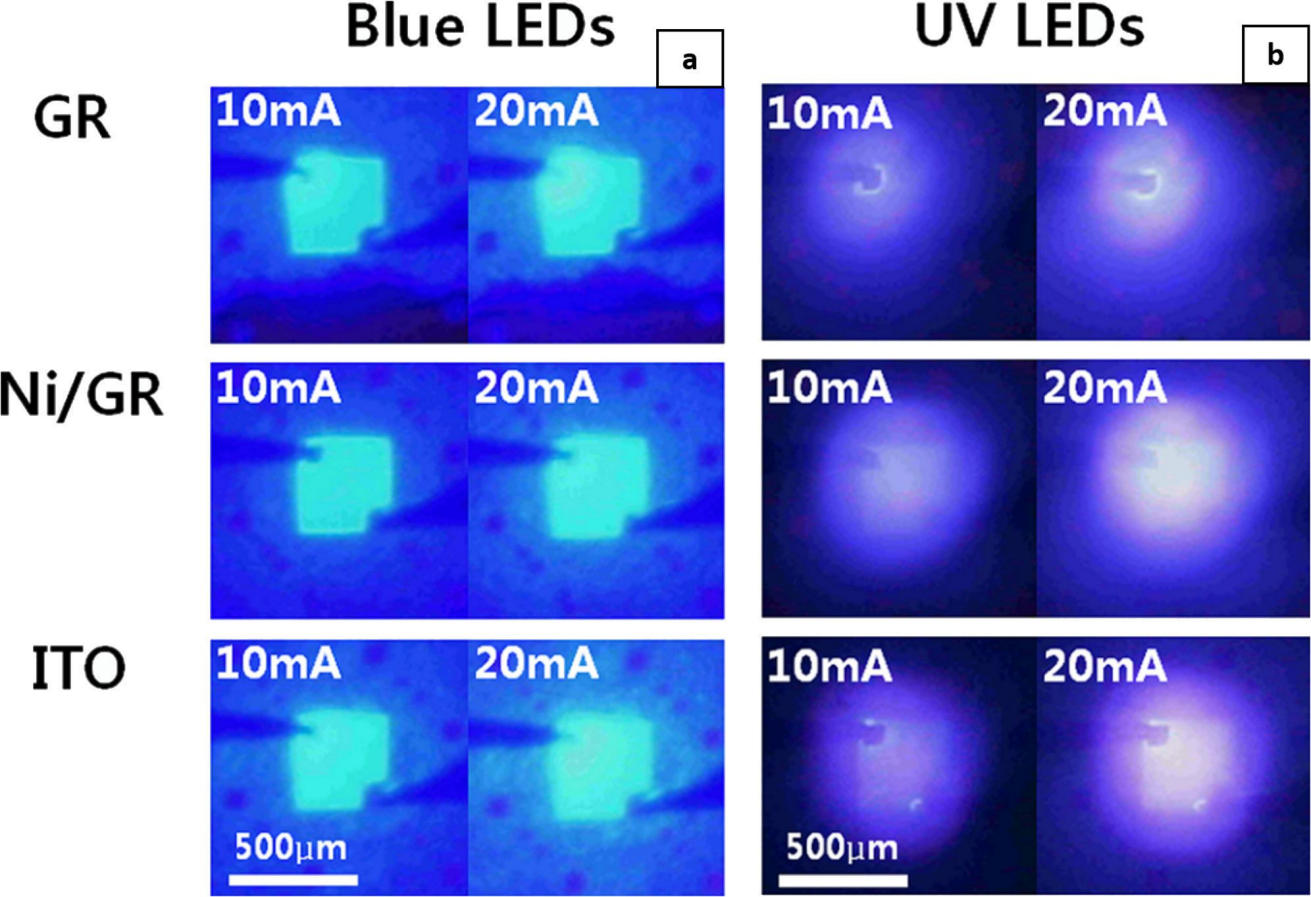
Optical images of light emission of (a) blue and (b) UV LED under different current-spreading layers (ITO, Ni on graphene, and graphene) at 1 and 20 mA current injections, respectively. Figure 3a and Figure 3b were adapted from [42], with the permission of AIP Publishing. This content is not subject to CC BY 4.0.

The anode can be supplemented with various metal nanostructures for improved performance. Figure 3 presents optical images of GaN-based blue LED and UV LED with different current-spreading layers (ITO, Ni on graphene, and graphene) [42]. For a particular injection current value, the turn-on voltages for blue LED recorded on various anodes were 3.5 (ITO), 6.2 (graphene), and 4.8 V (Ni on graphene). However, for UV LED, the observed operating voltage reduced significantly from 13.2 (graphene) to 7.1 V (Ni on graphene). Even though the ITO anode remained superior, the Ni–graphene nanocomposite nevertheless displayed 83% of the EL intensity of ITO. Furthermore, a decrease in sheet resistance (from 500 to 30 Ω) when Ag nanowires are added to graphene increases the ability of graphene to function as a current-spreading layer [43].

Equivalent to Ag, SPR of AuNP can also be applied to the anode in order to enhance the overall EL of the LED. Both of these MNP tend to increase the quantum efficiency of the LED. However, owing to the differences in the ranges of their SPR energies, different emission wavelengths are enhanced. In general, SPR of AgNP favors the quantum efficiency of the blue emission. On the other hand, Tanaka et al. reported that the SPR of AuNP positively influences the quantum efficiency of the red emission [44]. In addition, the shape of the MNP exhibiting SPR also plays a crucial role in the enhancement of the wavelength emanating from the LED. They studied the effect of Au nanorods (AuNR) and nanospheres (AuNS) in the device configuration of glass/ITO/AuNR and AuNS/CuPc/Alq_3_ + DCM/Alq_3_/LiF/Al red LED. The SPR absorption of Au nanorods and nanospheres were approx. 650 and 520 nm, respectively. Therefore, Au nanorods were able to enhance the red emission from the LED with an EQE of 6.8 × 10^−4^ at 10 V compared to the device with Au nanospheres (EQE = 2.4 × 10^−4^).

**Figure 4:**
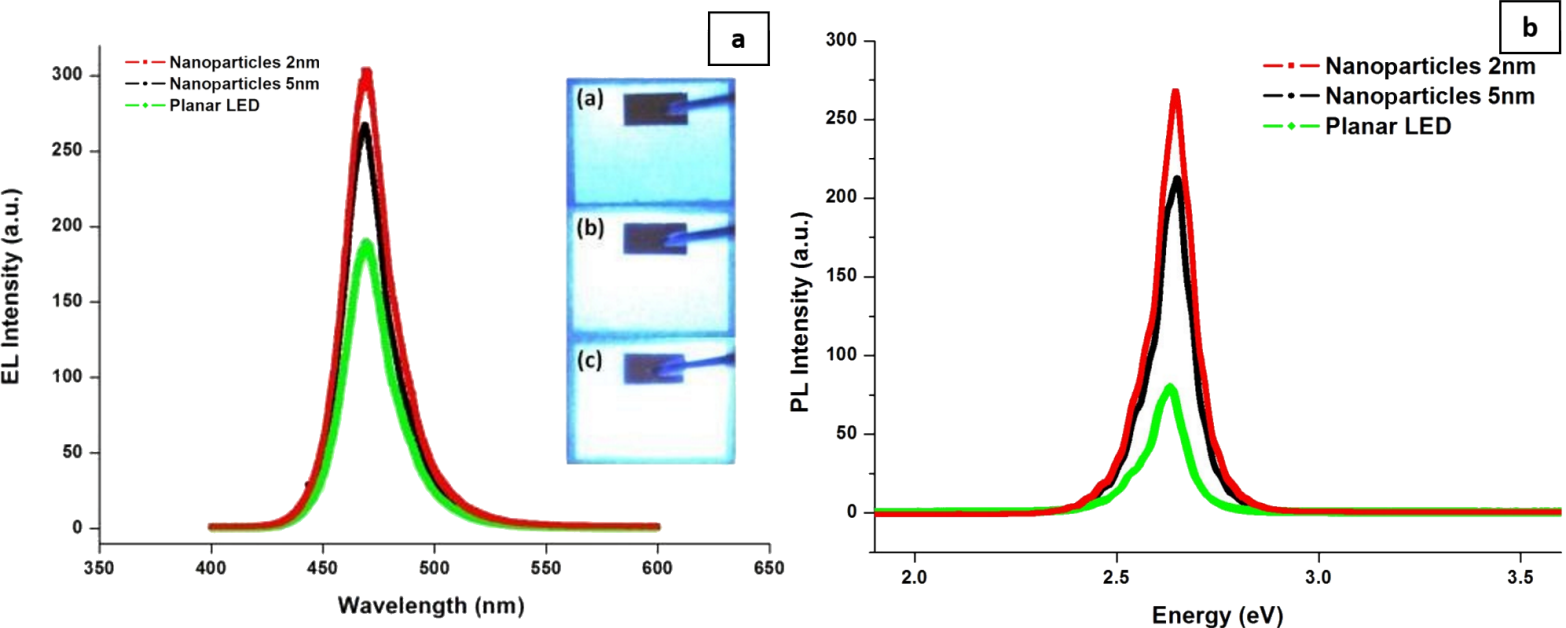
(a) EL spectra of LED having 2 and 5 nm Au–CNT system with an injection current of 100 mA measured at room temperature, using a planar LED as reference. The inset shows the optical images (a) without AuNP, (b) 5 nm Au–CNT system, and (c) 2 nm Au–CNT system. (b) Room temperature PL spectra of GaN LED with a 2 and 5 nm Au–CNT system and a planar LED as reference. Figure 4a and Figure 4b were adapted from [45] (“Enhanced optical output power of blue light-emitting diodes with quasi-aligned gold nanoparticles“, © 2014 Y. Jin et al., distributed under the terms of the Creative Commons Attribution 2.0 International License, https://creativecommons.org/licenses/by/2.0/).

In addition to the shape of AuNP, their size also influences the performance of LED. To that end, AuNP of diameters 2 and 5 nm were combined with CNT and deposited onto a p-GaN-based anode in a multiquantum-well LED [45]. The EL spectra of these devices at current injections of 100 mA have shown clear enhancements for both types of AuNP in Figure 4a and Figure 4b. Surface plasmon resonance absorbance tends to blueshift with decreasing sizes of the NP. Therefore, the 2 nm AuNP were able to enhance the blue emission from the LED. A similar effect was also observed with super-aligned carbon nanotubes (SACNT) decorated with AuNP (2 nm) [46]. The hybrid material was employed as a current-spreading layer in AlGaInP LED. At a current injection of 2 mA, a decrease in the forward voltage from 2.18 (without SACNT) to 2.03 V (with Au-coated SACNT) was noted. A decrease in the forward voltage and an increase in the optical power (≈10%) also indicated a reduced sheet resistance. In addition, AlGaInP LED emanating red emissions in the range of 560–650 nm are boosted via SPR of AuNP by varying the AuNP size as explained above. Table 3 provides a list of various LED properties as a function of carbon-based nanomaterials and plasmonic NP incorporated into different layers.

**Table 3:** EQE, turn-on voltage, and maximum luminance of various LED with carbon nanostructures, plasmonic, and metal oxide NP in the device layers.

LED	Emissive layer	Graphene/CNT	Plasmonic NP/metal oxide NP	EQE (%)	Turn-on voltage (V)	Maximum luminance (cd/m^2^)	Ref.

OLED	Alq_3_	MWNT in HIL	—	—	8.3	6800	[18]
OLED	Alq_3_	SWNT as the anode	—	—	6.6	2800	[21]
OLED	Alq_3_ + DCM	—	Au in the anode	0.00068	—	—	[44]
OLED	Alq_3_ + DCM	SWNT in HTL	—	0.05	—	—	[47]
OLED	Alq_3_	—	Ag in HIL	—	5	782	[48]
OLED	Alq_3_	—	Ag in HIL	2.08	5	4000	[49]
OLED	Alq_3_	—	Au/Ag in the anode/HTL	—	3/5	9000/8665	[50]
OLED	Alq_3_	graphene oxide as HIL	Au in HIL	0.77	3	3520	[51]
OLED	Ir(ppy)_2_(acac)	—	Ag in ZnO EIL	21.2	3.5	—	[52]
OLED	SWNT	SWNT as EML	—	—	1.3	—	[53]
OLED	MEH-PPV	graphene in EML	—	—	6	480	[54]
OLED	MEH-PPV	—	TiO_2_ in HIL	—	2.5	—	[55]
Inorganic LED	AlGaInP	graphene in anode	—	—	1.5	—	[40]
Inorganic LED	GaN	graphene as the anode	Ni in the anode	—	4.8/7.1	—	[42]
Inorganic LED	InGaN/AlGaN	graphene as the anode	Ag in the anode	—	4.48	—	[43]
Inorganic LED	GaN	—	Au in the anode	—	2.7	—	[45]
QLED	CdSe/ZnS	—	Ga doped ZnO as ETL	—	3	44000	[56]
QLED	MgZnO	—	Au in ZnO EIL	4.626	6	10206	[57]
QLED	Green QD	—	Yb and Ag in the cathode	9.8	2.2	—	[58]
HyLED	CdSe	—	Ag in the cathode	0.52	—	2000	[59]
HyLED	F8BT	—	ZnO as ETL	—	2	13100	[60]
HyLED	Imidazole	—	Ag-doped ZnO as EIL	15.2	1.75	10982	[61]

**Figure 5:**
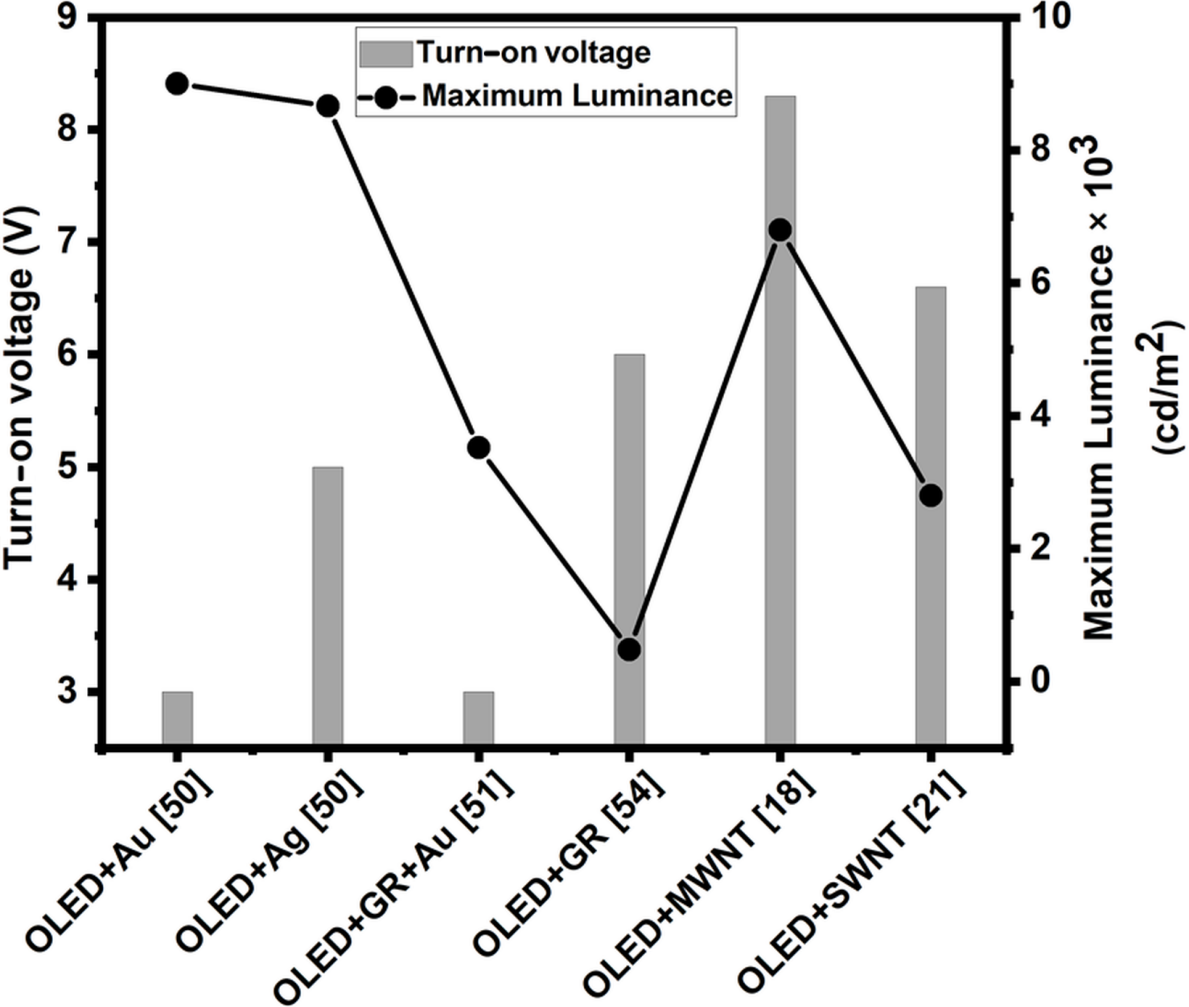
Comparison of turn-on voltage and maximum luminance of OLED with AgNP, AuNP, graphene, MWNT, and SWNT in the device structure.

Based on the data listed in Table 3, Figure 5 was plotted to compare the turn-on voltage and maximum luminance values of different OLED in order to evaluate the effectiveness of plasmonic nanoparticles and carbon-based nanostructures in the device architecture. Six types of OLED with AgNP, AuNP, GR, MWNT, SWNT, and GR + AuNP in the device structure were compared. In general, plasmonic nanoparticle-based OLED demonstrate lower turn-on voltages and higher luminance compared to the graphene or CNT-based ones. However, CNT and graphene tend to provide better thermal management and electrical properties; therefore, their integration into LED is of growing importance. Out of the three OLED-incorporating carbon-based nanostructures, OLED + GR possessed the lowest turn-on voltage; however, the luminance was rather low. Nevertheless, combing graphene with plasmonic nanoparticles (OLED + GR + Au) demonstrated a low turn-on voltage, equivalent to OLED + Au but a much lower luminance. Therefore, an ideal ratio between carbon-based nanostructures and plasmonic nanoparticles in OLED needs to be determined.

### Enhancing hole mobility: hole injection and transport layers

The optimization of charge carrier injection also consists of reducing the driving voltage of the LED. For achieving this, holes should be readily injected from the high-work-function anode surface (e.g., ITO, SWNT), while the electrons should be injected from the low-work-function metal cathode surface. Therefore, HTL should possess excellent charge mobility and maintain morphological stability. Moreover, it should have an appropriate highest occupied molecular level (HOMO), ensuring a low energy barrier for hole injection from the anode into the EML. A suitable lowest unoccupied molecular level (LUMO) should be capable of blocking electron injection from the EML to the HTL. Thus, HTL play a very important role in OLED and HyLED.

With regards to carbon-based nanocomposites, Kim et al. have synthesized SWNT–PVK nanocomposites for HTL in OLED with the configuration ITO/PEDOT:PSS/SWNT–PVK nanocomposite/DCM-doped Alq_3_/Li:Al [47]. An efficient electron transport was also obtained from the Li:Al cathode (with work function of 2.9 eV) to the Alq_3_ layer due to its higher LUMO level (3.2 eV) in comparison to the Li:Al work function. Therefore, this results in an efficient electron–hole recombination in the DCM-based EML. Moreover, the EQE of the device with SWNT–PVK with 0.2 wt % CNT shows twice the enhancement as compared to the device without SWNT. In addition, MWNT also enhance hole-injection capabilities [62]. Certain concentrations of MWNT were studied in PET/ITO/MWNT–PEDOT:PSS (80 nm)/Alq_3_/Al (40 nm) devices. At particular MWNT concentrations, higher current values (10 mA) and lower turn-on voltages (5 V) were noted. An optimal MWNT concentration of 0.6 wt % was deduced at which the EL intensity increased and the operating voltage decreased by a significant amount. However, a major concern regarding the reduction of the electron-blocking capability of PEDOT:PSS due to the addition of metallic MWNT needed further insight. Therefore, additional work by the same group [18] reported that the addition of MWNT in appropriate concentrations (0.4 wt %) improved the hole-injecting ability of PEDOT:PSS. Incorporation of 0.005 wt % of SWNT in PVK HTL resulted in 55% increment in hole mobility, which was initially 2.5 × 10^−6^ cm^2^/V·s [63].

Other carbon-based nanostructures for HTL include graphene oxide. However, an optimum thickness of graphene oxide is required based on the device configuration. Shi et al. obtained similar results with an excellent luminance of 53000 cd/m^2^, demonstrating its explicit applicability in flexible OLED [64]. Combinations of graphene oxide with polymers and metal oxides have also been evaluated. Lin et al. obtained an optimum concentration of 0.03 wt % of graphene in PEDOT:PSS [65]. A combination of MoS_2_ and graphene sheets has also displayed good HIL tendencies due to the high surface coverage, work function, and low LUMO levels of graphene [66].

With regards to SPR of MNP, green emission enhancement in OLED has also been reported owing to the SPR of AgNP with an average size of 80 nm [48]. Silver nanoparticles were embedded in a PEDOT:PSS layer within the follwing device configuration: ITO (150 nm)/PEDOT:PSS (60 ± 10 nm)/AgNP/Alq_3_ (100 nm)/LiF (1 nm)/Al (100 nm). The PL emission intensity at 535 nm from the device with AgNP increased by 30% compared to the device without AgNP. Choi et al. have used a scattering layer of AgNP in order to study the SPR effect, which increased the EQE by 24% in the flexible OLED [49].

**Figure 6:**
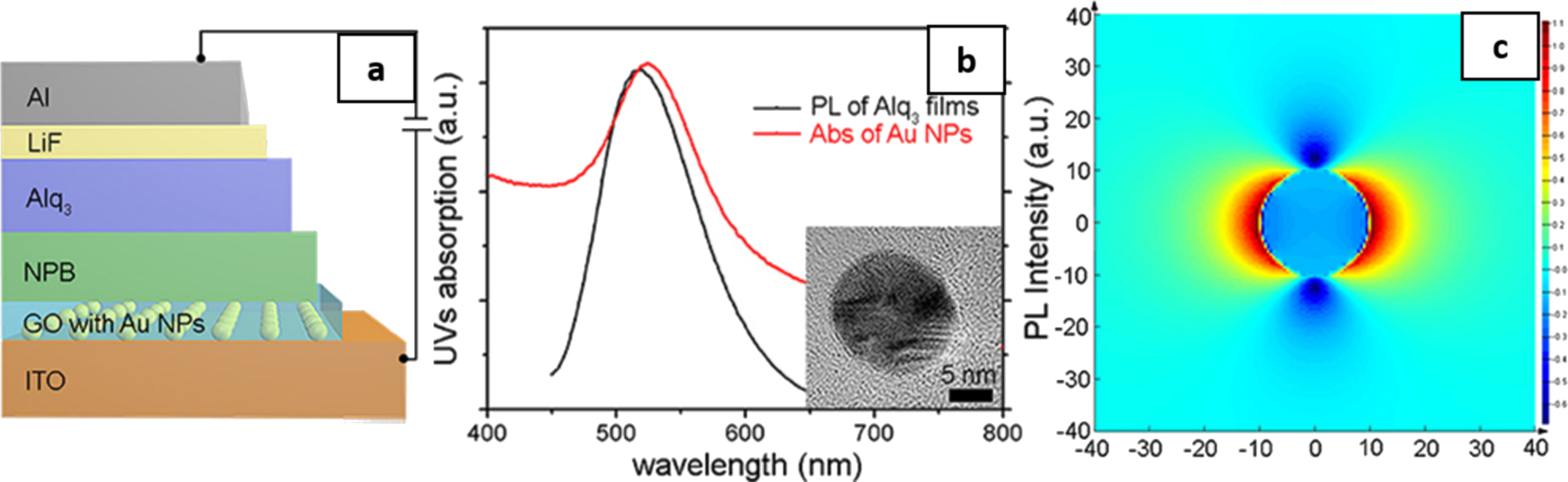
(a) OLED device structure with graphene oxide–AuNP inserted between ITO anode and NPB HTL. (b) The absorption spectrum of AuNP and PL emission spectrum of Alq_3_ on ITO glass. The inset shows the TEM image of AuNP with a particle size of 20 nm. (c) The electromagnetic field distribution around AuNP with 20 nm diameter simulated by finite-difference time-domain modelling. Figure 6a–c was adapted from [51] (© 2018 J. Feng et al., distributed under the terms of the Creative Commons Attribution 4.0 International License, https://creativecommons.org/licenses/by/4.0/).

Au and AgNP have also been embedded at the interface of the anode and the HTL in OLED [50]. Depending on the surface coverage of the plasmonic NP, an increment in the current density by a factor of two as compared to a device without plasmonic NP has been observed. The presence of Au and Ag reduces the work function of ITO due to the formation of dipoles at the interface and, therefore, reduces the hole injection barrier, which in turn creates a more efficient transfer to the HTL. In the device configuration of Figure 6a, a graphene oxide–Au nanocomposite HIL inserted between ITO and NPB was used to enhance the EL of Alq_3_-based OLED [51]. The correlation between the wavelength absorbed (≈540 nm) by AuNP and the PL emission of the Alq_3_ thin film (≈530 nm) implies a suitable plasmonic coupling with the NPB HTL (Figure 6b). In Figure 6c, the SPR coupling radius for a 20 nm AuNP was estimated to be ≈10 nm. This implies that the excitonic recombination occurs very close to the NPB and Alq_3_ interface. The study reports that the devices with 10% graphene oxide–Au nanocomposites show approx. 45% improvement in their maximum luminance, maximum current efficiency, and maximum EQE. Similar results were also obtained for AgNP in other studies [67].

### Enhancing electron mobility: electron transport and injection layers

Similar to HTL and HIL, ETL and EIL play very crucial roles in optimizing charge carrier injection in OLED and HyLED. The latter follows an inverted OLED architecture (i.e., the EIL is in contact with the cathode followed by ETL). In order to enhance the OLED performance, ETL should have a high reduction potential and appropriate HOMO and LUMO values relative to the p-type emitter and HTL [68]. Other factors affecting their performance include a high electron mobility, an amorphous morphology, a high glass transition temperature, and the ability to be deposited as a uniform thin film. Some of the commonly used polymers and metal oxides for ETL are PBD, PBD-PMMA, BND, ZnO, SnO_2_, and TiO_2_ [69–71].

**Figure 7:**
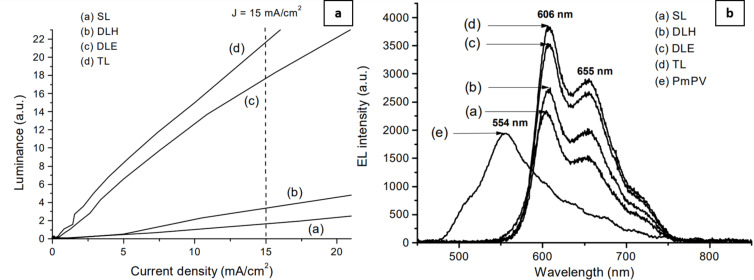
(a) Luminance as a function of current density and (b) EL spectrum for a MWNT concentration of 8 wt % for all the devices. Figure 7a and Figure 7b were adapted from [19], with the permission of AIP Publishing. This content is not subject to CC BY 4.0.

Improvements in the device performance have been reported, when using polymer–MWNT nanocomposite-based ETL. For example, Fournet et al. have investigated the role of MWNT by varying their concentration from 0–32% in PmPV [19]. They investigated a series of devices with configurations ITO/M3EH-PPV/Al (SL), ITO/PVK/M3EH-PPV/Al (DLH), ITO/M3EH-PPV/MWNT–PmPV/Al (DLE), and ITO/PVK(HTL)/M3EH-PPV(EML)/MWNT–PmPV (ETL)/Al (TL). The TL devices with 8 wt % of MWNT (Figure 7) present the best results in terms of luminance and EL emission. In addition, the electron conductivity of the device is increased by four orders of magnitude. Concerning graphene–polymer nanocomposites, Choudhary et al. have inserted MoS_2_ and graphene oxide NP into a polyaniline ETL [72]. The conductivity of the nanocomposite was enhanced by 185% as compared to pure polyaniline. Further, the role of carbon quantum dots (CQD) in the ETL has also been examined in the device ITO/PEDOT:PSS/PFO/CQD/LiF/Al [73]. The turn-on voltage with CQD in the device configuration reduced from 8 to 6 V with enhancements in performance, efficiency, and lifetime compared to a pristine PFO device. In a work by Park et al., a remarkable enhancement in the electron mobility of ZnO ETL was reported on dispersing 0.08 wt % of n-doped CNT on its surface [74]. The effective electron mobility of the device without CNT was 1.5 × 10^−6^ cm^2^/V·s. In contrast, the device with n-doped CNT showed an effective electron mobility of 7.0 × 10^−6^ cm^2^/V·s, which corresponds to a five-fold improvement.

**Figure 8:**
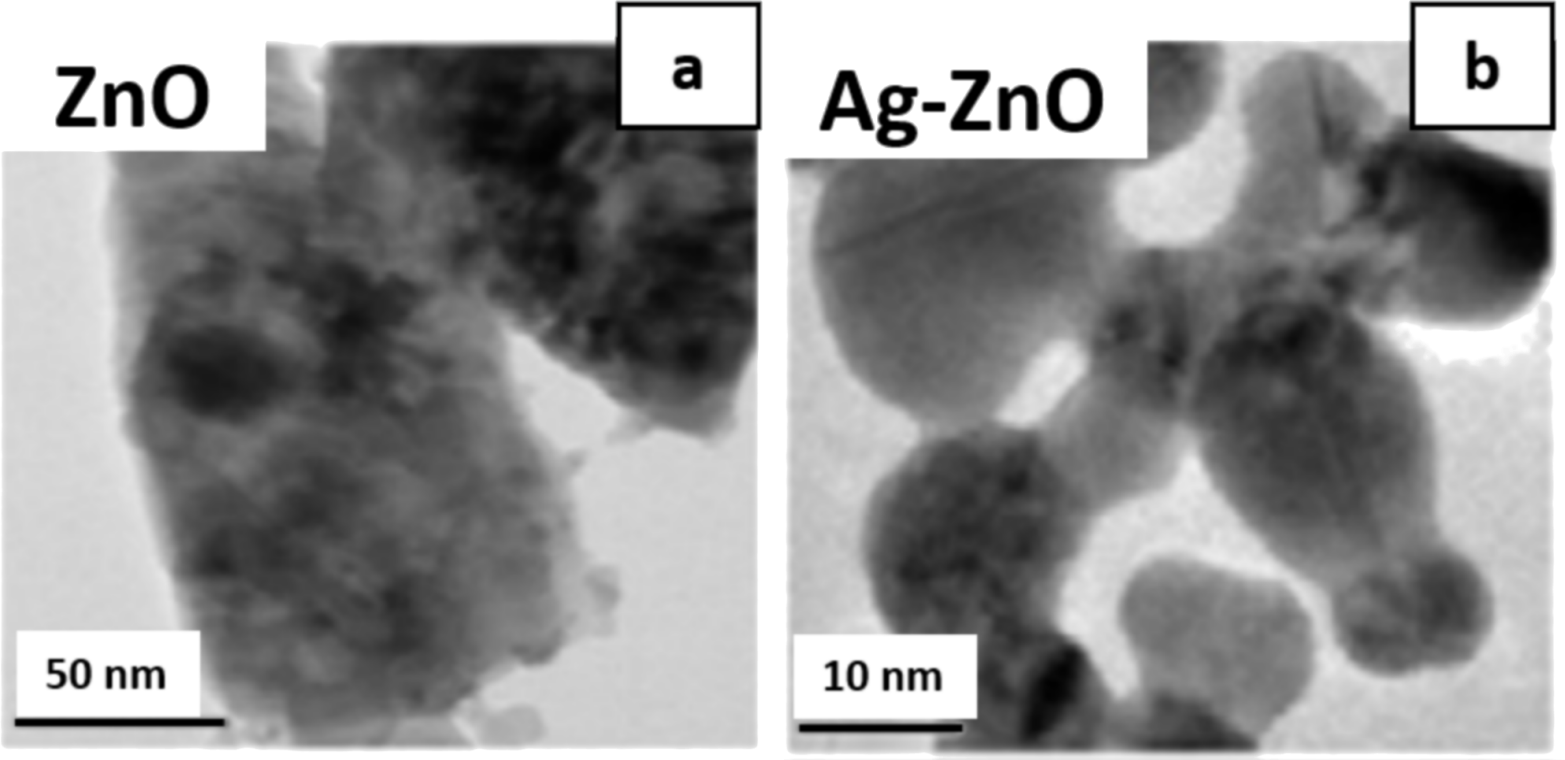
Transmission electron microscopy images for (a) pristine ZnO and (b) 2–3% Ag-doped ZnO. Figure 8a and Figure 8b were adapted from [61], J. Photochem. Photobiol. A, vol. 325, by J. Jayabharathi; A. Prabhakaran; V. Thanikachalam; M. Sundharesan,“Hybrid organic-inorganic light-emitting diodes: Effect of Ag-doped ZnO”, pages 88–96, Copyright (2016), with permission from Elsevier. This content is not subject to CC BY 4.0.

Incorporation of plasmonic NP in the ETL or EIL has further manifested a significant enhancement in the overall performance of LED. For example, Zhou et al. have used Ag-modified ZnO NP film as EIL in inverted fluorescent OLED (IFOLED) and inverted phosphorescent OLED (IPOLED) [52]. As a result, the IFOLED and IPOLED show very high current efficiencies of 8.4 and 95.3 cd/A and EQE of 4% and 21%, respectively, at a current density of 20 mA/cm^2^. Similarly, the roles of Ag-doped ZnO and pristine ZnO as EIL in the imidazole organic EML-based HyLED were compared [61]. The transmission electron microscopy (TEM) images of Figure 8 illustrate that the pristine ZnO NP are larger (≈50 nm) than the Ag-doped ZnO (≈15 nm). In addition, the device with 2–3% Ag-doped ZnO EIL shows maximum luminance of 10982 cd/m^2^, maximum current efficiency of 41.0 cd/A, and EQE of 15.2%. The addition of AgNP to PBD-based ETL enhances the green emission [75]. Kandulna et al. have addressed the effect of plasmonic NP on hybrid metal oxide–polymer nanocomposites [76]. They investigated Ag-doped ZnO (Ag:ZnO) and Ag:ZnO/PMMA nanocomposites for ETL applications in OLED. The pristine Ag:ZnO has an average particle size of ≈57 nm and the PMMA capping layer was ≈8 nm. Consequently, an increased rate of electron–hole recombination and an enhanced current density of ≈85% were obtained for optimized 10% Ag:ZnO/PMMA nanocomposite as compared to pristine Ag:ZnO.

### Enhancing the emissive layer

The emissive layer is also known as the active luminescent region of an LED, where electron–hole recombination occurs. Light-emitting diodes and OLED are capable of emitting in the entire visible spectrum: GaAsP (red), GaN (green, blue/green), PPV (yellow/green), MEH-PPV and BCHA-PPV (yellow/orange), PPE-PPV (blue), and MDMO-PPV (red) [77–84]. Among these polymers, PPV appears to be the most popular light-emitting polymer. Burroughes et al. have reported on the first OLED using PPV that produces a yellow/green emission [80]. However, PPV has drawbacks, such as low-intensity PL emission and a high conversion temperature. Nevertheless, the derived versions of PPV, such as MEH-PPV, in which the emission shifts to orange from yellow/green in the unsubstituted PPV polymer, show improved properties.

More recently, QD-based LED have shown an increase in the overall performance and longer lifetime values than OLED [85–89]. Furthermore, facile tuning of the emission wavelength by adjusting the particle size, their low fabrication cost, and saturated colors with a narrow bandwidth of EL (full width at half maximum ≈30 nm) have made the QLED attractive [90–91]. In effect, owing to the quantum confinement of CdSe QD, a decrease in particle size blueshifts the emission. Thus, their emission can be tuned to the entire visible spectrum by varying their sizes [92]. Generally, a QD-based EML is sandwiched between a polymer-based ETL and HTL.

Carbon-based nanomaterials such as SWNT have also been investigated as EML in OLED. Firstly, a SWNT p–n junction on a Si substrate has been investigated by Lee et al. [93]. Further, Mueller et al., with some modifications to the design of Lee et al., were able to produce a significantly narrower (≈35 meV) EL spectrum in the red region [17]. Wang et al. improved the device design of Mueller et al. by using a polymer layer of PMMA instead of split gates [53]. The device of Wang et al. has several advantages: it is doping-free and cost-effective, it can be operated using a single bias, and it emanates a narrow EL spectrum of ≈30 meV. Similarly, white light LED can also consist of CQD at the EML along with metal oxides in the charge transport layers [94]. In addition, a maximum EQE of 0.083% has been obtained from CQD-based devices with configuration ITO/PEDOT:PSS (40 nm)/CQD (20 nm)/TPBI (40 nm)/LiF/Al [95]. These results clearly suggest that CQD are potential phosphors in the fabrication of white LED [96–100].

Prasad et al. have incorporated graphene nanosheets in MEH-PPV in order to evaluate the optimum quantity (0–0.1 wt %) for the luminance of the device [54]. They observed an approximate six-fold increase in the PL emission for 0.005 wt % of graphene nanosheets. The reason for the increased PL emission is attributed to the higher charge carrier mobility in graphene nanostructures, which balances out the charge carrier concentration in the EML.

**Figure 9:**
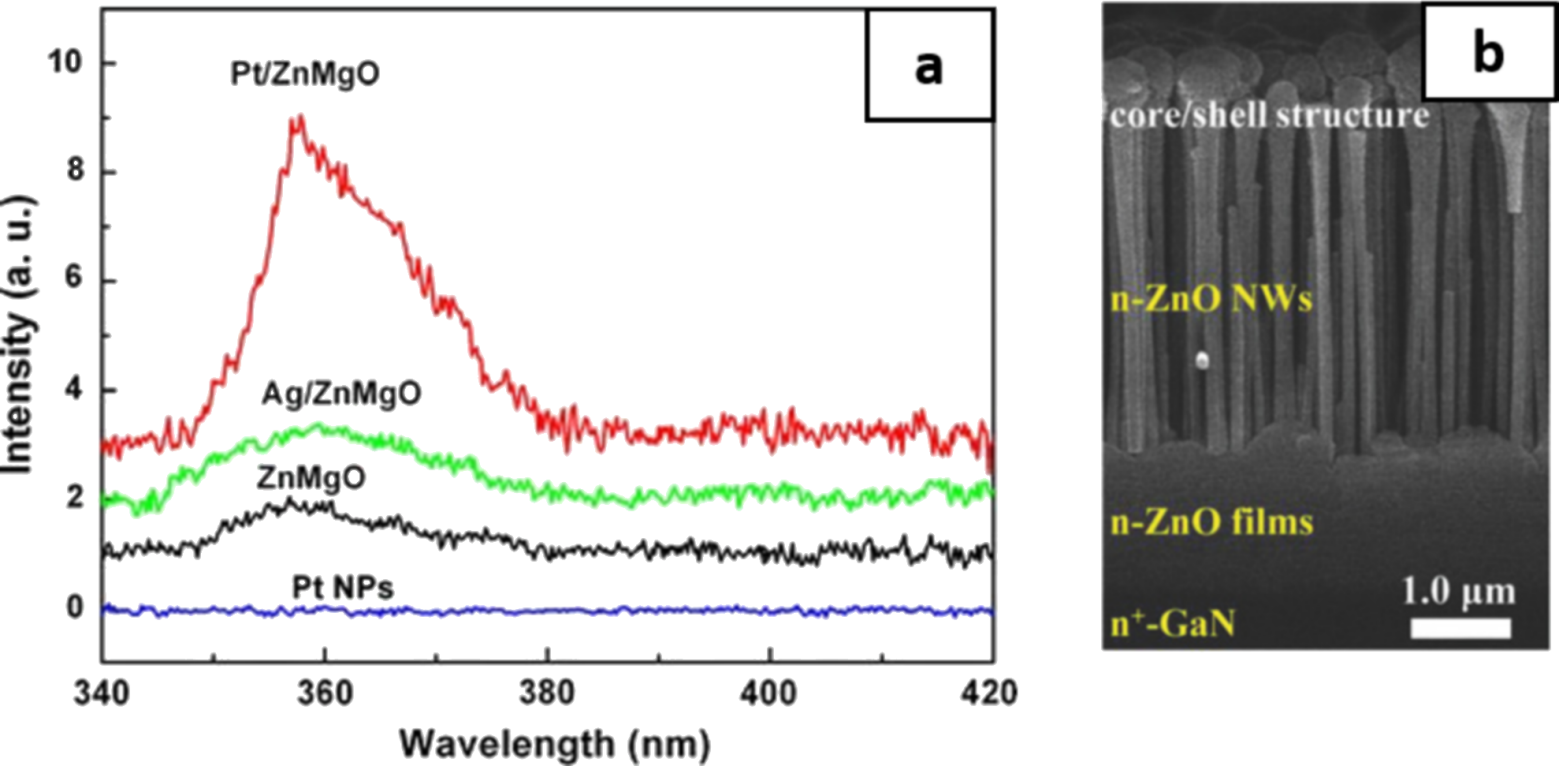
(a) Room temperature PL spectra of ZnMgO, Pt/ZnMgO, Ag/ZnMgO, and PtNP. A six-fold and a two-fold enhancement in the PL emission is observed for Pt/ZnMgO and Ag/ZnMgO, respectively. (b) Cross-sectional SEM image of the device architecture. Figure 9a was adapted from [104] (“Localized-Surface-Plasmon Enhanced the 357 nm Forward Emission from ZnMgO Films Capped by Pt Nanoparticles“, © 2009 J. B. You et al., distributed under the terms of the Creative Commons Attribution 2.0 International License, https://creativecommons.org/licenses/by/2.0/). Figure 9b was adapted from [57], Z. Shi et al., “Localized Surface Plasmon Enhanced All-Inorganic Perovskite Quantum Dot Light-Emitting Diodes Based on Coaxial Core/Shell Heterojunction Architecture”, Adv. Funct. Mater., with permission from John Wiley and Sons. Copyright © 2018 WILEY-VCH Verlag GmbH & Co. KGaA, Weinheim. This content is not subject to CC BY 4.0.

InGaN-based LED have also benefited from the incorporation of AgNP in the EML. Consequently, a 14-fold PL enhancement and a seven-fold internal quantum efficiency have been reported [101]. A similar work reported an increase in the optical output power by 32% for an injection current of 100 mA [102]. By incorporating AgNP in F8BT, the current efficiency increased by a factor of 75, along with an approximate two-fold enhancement in the EL intensity [103]. You et al. have examined the PL emission from a ZnMgO alloy with a surface-capping of PtNP with thickness values ranging from 2 to 8 nm and with AgNP of 6 nm of thickness [104]. A six-fold and a two–fold enhancement in the PL emission has been observed by capping the ZnMgO alloy with Pt and AgNP, respectively. Figure 9a is the PL spectra of the ZnMgO, Pt/ZnMgO, Ag/ZnMgO and PtNP at room temperature showing a clear increase in the PL emission. Besides Ag nanorods and nanospheres, other plasmonic nanostructures, such as Ag nanocubes and nanostars tend to enhance LED properties owing to their sharp facets and edges [105–106]. Yu et al. have incorporated Ag nanocube core coated with SiO_2_ shell in the EML of blue light-emitting diode [107]. A very high improvement in the current efficiency compared to the device without Ag nanocubes was noted. Shi et al. in a report, have introduced AuNP in the MgZnO QD layer with a device structure of p-NiO/CsPbBr_3_/MgZnO/AuNP/n-ZnO film/n-GaN (Figure 9b) [57]. They further optimized the thickness of the MgZnO layer to 10 nm in order to control the distance between the CsPbBr_3_ layer and AuNP. The EQE increased from 3 to 4.5% as the thickness of the MgZnO layer increased from 0 (no MgZnO layer) to 10 nm. The enhancement in the EQE and overall device performance has been associated with the spontaneous emission rate induced by SPR coupling when an optimal distance of separation exists between the plasmonic and active layers.

### New possibilities for cathode materials

The performance parameters of OLED, for example, turn-on voltage, operating voltage, current/power efficiency, and lifetime are strongly dependent on the physical parameters of the cathode material [108]. A cathode material should generally have a low work function as compared to the adjacent layer (generally ETL) in the device. The efficiency of electron transport depends on the energy difference between the Fermi level of the cathode material and the LUMO level of the adjacent material in which electrons have to be transported. Therefore, decreasing the energy barrier between the cathode and the adjacent layer can increase the electron injection efficiency and, hence, the device efficiency. Despite the high work function of Al (4.31 eV), it is among the most suitable cathode materials due to its high stability in ambient conditions. Alkali and alkali-rare earth metals have lower work function (2.8–3.7 eV) but are unstable in ambient conditions.

There are a few examples where plasmonic NP are used at the cathode to enhance the overall emission of the LED. Plasmonic AgNP have been inserted in Al (150 nm)/LiF (1 nm) cathode layer and a PL enhancement was observed [109]. In fact, the presence of AgNP tuned the carrier injection rate between cathode and Alq_3_ EML. Carbon nanomaterials, such as graphene and SWNT present interesting alternatives to overcome the drawbacks of metal and metal nanocomposite cathodes in OLED. Klain et al. have reported one possibility by doping graphene with n-type Ca [110]. The 1 nm Ca layer deposited by evaporation onto the surface of graphene reduced its work function by nearly 1 eV. Furthermore, the *I*–*V* characteristics of the device with Ca-doped graphene/Alq_3_/Ag manifest a two-fold increase in current (at 2 V bias) as compared to only graphene. Similarly, Chang et al. have doped graphene with n-type CsF or Cs_2_CO_3_ on a SiO_2_ substrate, thus reducing the work function of graphene from 4.4 to 3.2 eV, which was used to fabricate blue light-emitting OLED with maximum brightness of 1034 cd/m^2^ [111]. On the other hand, very few works have been reported where SWNT are employed as cathodes in fully transparent OLED. Chien et al. have achieved highly efficient electron injection from SWNT-based films by modifying the surface of SWNT with a thin layer of Pd/Al/LiF while maintaining the transparency of the layer [112]. However, the device was ineffective as no emission was visible and the turn-on voltage was unusually low. The lack of emission was attributed to the charge unbalance in the EML due to high electron injection from the SWNT. Further efforts towards balancing electron–hole injection in the EML could also result in light emission from such devices. This could be brought about by optimizing the quantity of SWNT, functionalizing them, and combining them with other polymers. Nevertheless, this work demonstrates that SWNT can be used as cathode materials in LED, if improved. The successful implementation of such an electrode would open new possibilities to fabricate fully transparent OLED.

## Conclusion

This article has provided an overview of the combination of various nanostructures that enhance the overall properties of various types of LED. In particular, CNT and MNP exhibiting SPR improve several characteristics of LED, such as lower turn-on voltages, higher EQE, and high luminance. The versatility of these nanomaterials lies in the fact that they can be accommodated into one or several layers of the LED. For example, carbon-based nanostructures can be added to the anode, hole injection, and transport layers as well as the EML. On the other hand, SWNT are still being tested as a viable cathode material. Depending upon the layer, these carbon-based nanostructures are able to enhance current spread, injection, and recombination lifetime values. Similarly, plasmonic NP, such as Au and Ag clearly enhance LED properties and can be integrated in all the layers of the device structure. Furthermore, incorporation of metal oxides, such as TiO_2_, ZnO, SnO_2_ in combination with Ag, Au, and PtNP has shown increment in the luminance and reduction in the operating voltages of the devices.

For several decades, inorganic LED based on III–V group semiconductors (i.e., GaAs, GaP, InGaAs, InGaP, GaN, InGaN, and AlGaInP) have been extensively studied and improved. More than 50% EQE for AlGaInP (red) and InGaN/GaN (blue/green) emitters has already been achieved with a future possibility of reaching up to 80%. Moreover, achievement in the device stability of up to 20000 h is remarkable for inorganic LED. However, these inorganic LED have poor color quality and they are fabricated with rare, costly, and non-flexible materials, which hinder their wide scale applications in wearables and display technologies. In addition, the self-heating of such structures is unavoidable. Carbon nanotubes and plasmonic NP exhibiting SPR have shown their superiority in enhancing the color quality of these inorganic LED by enhancing the PL, EL, current efficiency, and density. Moreover, graphene and SWNT incorporated into the anode have opened opportunities for thermal management in flexible inorganic LED.

Currently, OLED and QLED are dominating the display market. Light sources in OLED mainly consist of Alq_3_ and derived version of PPV polymer, whereas QD based on Cd, Zn, Se, and S are mainly used for QLED. Both technologies have many advantages over traditional inorganic LED and liquid crystal displays in terms of low power consumption, wider view angle, higher resolution and cost-effectiveness. However, device degradation is common in both OLED and QLED due to charge accumulation in the layers. In the case of red QLED, HTL is rather stable and the device degradation rate is therefore low. On the other hand, in the blue QLED, their poor lifetime is caused by the quicker degradation of ETL. A similar degradation in OLED has also been observed but mainly in the HTL; the poor hole injection ability of polymers is also a cause for concern. From a commercial point of view, the present OLED displays are expensive as compared to QLED displays consisting of Cd-based QD. Graphene and CNT could be viable options in slowing or circumventing the degradation of HIL in OLED and QLED when combined with polymer nanocomposites.

In order to enhance the color purity of the OLED-based displays, SWNT combined with an emissive polymer tend to increase the luminance of the device and thereby achieve a higher color saturation. Nevertheless, SWNT display an emission spectrum with full width at half maximum of approximately 30 nm, which is very close to the color saturation of QLED displays (≈25 nm). The blue/green emission from InGaN LED, ZnO NP, and OLED is enhanced through the SPR effect of AgNP, which in turn could counteract the high external luminance. Thus, the low brightness and short operation lifetime of blue QLED could be improved by using CNT–polymer or CNT–metal oxide nanocomposites as ETL and incorporating AgNP in this layer. Further strategies to counteract the outdoor luminance of 7000 cd/m^2^ could also include a graphene oxide HIL, in which a luminance of 56000 cd/m^2^ has been reported in such OLED. Doped ZnO in the ETL is also a suitable candidate for high-luminance blue OLED. Since the degradation of these nanocomposites is relatively slow as compared to pure CNT, they could therefore provide a workable pathway to enhance the lifetime of the blue QLED. Moreover, due to the localized SPR of AgNP the brightness of blue QLED can also be improved significantly. Enhancement of the red luminescence has been possible by the addition of AuNP. Enhancing the green luminescence has not been widely studied in OLED and QLED. However, AgNP has the potential to enhance the green emission through the engineering of its shape and size in order to modify their SPR properties.

OLED- and QLED-based displays are commercially available. Nonetheless, cost-effective and better quality displays are constantly being developed. The combination of various polymers with inorganic components or HyLED could very well fulfill the future needs of the display and lighting market. Even though HyLED is commercially available for lighting applications, further work still needs to be carried out in order to implement them in display technologies. Nevertheless, a combination of organic, inorganic, carbon nanostructures, and plasmonic NP could very well pave the way for future display technologies.
